# Overnight Resting of PBMC Changes Functional Signatures of Antigen Specific T- Cell Responses: Impact for Immune Monitoring within Clinical Trials

**DOI:** 10.1371/journal.pone.0076215

**Published:** 2013-10-11

**Authors:** Sarah Kutscher, Claudia J. Dembek, Simone Deckert, Carolina Russo, Nina Körber, Johannes R. Bogner, Fabian Geisler, Andreas Umgelter, Michael Neuenhahn, Julia Albrecht, Antonio Cosma, Ulrike Protzer, Tanja Bauer

**Affiliations:** 1 Institute of Virology, Technische Universität München/Helmholtz Zentrum München, Munich, Germany; 2 Cooperation Group ‘Immune Monitoring’, Helmholtz Zentrum München, Munich, Germany; 3 Department of Infectious Diseases/Med. Klinik und Poliklinik, University Hospital of Munich/Ludwig Maximilians Universität, Munich, Germany; 4 Department of Internal Medicine II, Klinikum rechts der Isar/Technische Universität München, Munich, Germany; 5 Institute of Medical Microbiology, Immunology and Hygiene, Technische Universität München, Munich, Germany; 6 Division of Immuno-Virology, CEA, Paris, France; 7 German Center for Infection Research (DZIF), Munich, Germany; Istanbul University, Turkey

## Abstract

Polyfunctional CD4 or CD8 T cells are proposed to represent a correlate of immune control for persistent viruses as well as for vaccine mediated protection against infection. A well-suited methodology to study complex functional phenotypes of antiviral T cells is the combined staining of intracellular cytokines and phenotypic marker expression using polychromatic flow cytometry. In this study we analyzed the effect of an overnight resting period at 37°C on the quantity and functionality of HIV-1, EBV, CMV, HBV and HCV specific CD4 and CD8 T-cell responses in a cohort of 21 individuals. We quantified total antigen specific T cells by multimer staining and used 10-color intracellular cytokine staining (ICS) to determine IFNγ, TNFα, IL2 and MIP1β production. After an overnight resting significantly higher numbers of functionally active T cells were detectable by ICS for all tested antigen specificities, whereas the total number of antigen specific T cells determined by multimer staining remained unchanged. Overnight resting shifted the quality of T-cell responses towards polyfunctionality and increased antigen sensitivity of T cells. Our data suggest that the observed effect is mediated by T cells rather than by antigen presenting cells. We conclude that overnight resting of PBMC prior to *ex vivo* analysis of antiviral T-cell responses represents an efficient method to increase sensitivity of ICS-based methods and has a prominent impact on the functional phenotype of T cells.

## Introduction

Antigen specific CD8 and CD4 T cells are a crucial component of antiviral immune responses known to play an important role in limiting viral replication and controlling virus-associated diseases [1]. Monitoring functional signatures of antiviral T-cell immunity is one prerequisite to identify correlates of immune protection in clinical trials aiming to develop innovative antiviral therapies (e.g. therapeutic vaccines) [2]. Here the primary objective of immune monitoring is to determine the efficacy of a vaccine to induce or boost a specific T-cell response. Failure of recent large-scale clinical trials investigating HIV-1 vaccine candidates pointed out that detailed monitoring of vaccine-induced T cells in early phases of the clinical development are essential to evaluate vaccine efficacy [3].

The detection of T-cell responses by immune assays has recently been included as primary endpoints for clinical trials but phenotypic and functional analysis of T cells in clinical monitoring settings has been difficult to establish as different types of assays have been used and both standardization and validation of immune biomarker assays have often been lacking. Intracellular cytokine staining (ICS), enzyme-linked immunospot (Elispot) assays and multimer staining are commonly used for monitoring of antigen specific immune responses. Polychromatic ICS assays are routinely used in several laboratories as they enable simultaneous characterization of phenotype and functional repertoire of antigen specific T cells [2]. Implementation of complex ICS assays to measure detailed T-cell functionality requires an accurate standardization of the experimental protocol, because minor differences in the procedure can have a profound effect on T-cell marker expression [4]–[6].

Post-thaw resting of cryopreserved Peripheral Blood Mononuclear Cells (PBMC) for several hours or overnight at 37°C prior to *ex vivo* antigenic restimulation is a common practice but there are also numerous studies where this procedure was not included in the protocol. Data on the effect of overnight resting on T-cell monitoring results are contradictory. Overnight resting of PBMC can reduce unspecific cytokine production [7] and increase functional avidity of T cells [8] but is reported not to affect detectable frequencies of antigen specific T cells [4]. So far it has not been studied for a broad repertoire of viral antigens whether resting of PBMC is a variable affecting monitoring of antiviral T-cell responses.

In the present study, we analyzed the effect of overnight resting on the quantity and the functional signature of CD8 and CD4 T cells specifically recognizing antigens of human immunodeficiency virus (HIV), Epstein-Barr virus (EBV), human cytomegalovirus (CMV), hepatitis B (HBV) or hepatitis C virus (HCV). We observed that overnight resting influenced the functional profile of antiviral CD8 and CD4 T cells *ex vivo* detectable by ICS for all tested antigen-specificities. Our results strongly suggest standardization of immune assays for monitoring virus specific T-cell responses, enabling comparison of data across clinical trial sites.

## Materials and Methods

Materials and methods are structured according to the Minimal Information About T cell Assays http://www.miataproject.org/ [9], [10].

### Samples

#### Patients

This study was approved by the local ethics committee at Technische Universität München. Prior to enrolment, each patient or healthy volunteer signed a written consent to participate to the trial according to the Helsinki Declaration of ethical guidelines.

The cohort consisted of nine HIV-1 infected, three HCV infected, three HBV infected and six healthy volunteers (mean age: 45 years, range 26 to 68; 11 male and 10 female). Healthy volunteers were seronegative for HIV and HCV, vaccinated against HBV, had no signs or symptoms of acute infections at the time of blood sampling and had mean EBV-titers of 343 U/ml (range 130 to 710 U/ml).

#### PBMC isolation, cryopreservation, storage and thawing

Heparinized whole blood was collected by venipuncture and stored or transported at room temperature. PBMC were processed within 8 hours by Ficoll density gradient centrifugation (Biochrom AG, Berlin, Germany) following our established SOP. PBMC were washed twice with RPMI 1640 medium (Lonza, Cologne, Germany) and counted using trypan blue staining. Cells were resuspended in equal aliquots of 4, 5 or 10×10^6^ cells in cold freezing medium, consisting of heat-inactivated fetal calf serum (FCS; Biochrom AG, Berlin, Germany) containing 10% DMSO (Sigma-Aldrich, Munich, Germany). PBMC were slowly frozen (−1°C/minute) using a controlled-grade freezing device (Nunc, Wiesbaden, Germany) and stored over night at −80°C before storage in a vapor–phase liquid nitrogen vessel for average 6 months. For use cryopreserved PBMC were rapidly thawed in a waterbath at 37°C and washed twice with RPMI1640 supplemented with 1% penicillin/streptomycin (PenStrep) (Gibco, Darmstadt Germany) and 10% FCS. Afterwards living PBMC were counted using trypan blue staining.

#### Overnight resting of PBMC

After thawing PBMC were either rested or used directly for ICS. For standard resting procedure, PBMC (2×10^6^ cells/ml/max 20×10^6^ cells) were resuspended in RPMI 1640 medium supplemented with 1% PenStrep and 10% FCS in a V-shaped 50 ml tube (Falcon BD, Heidelberg, Germany) and incubated for 18 h at 37°C in a humidified atmosphere at 5% CO_2_. In order to allow gas exchange the tube cap was loosened.

#### Peptides and multimers

For *ex vivo* restimulation of PBMC in intracellular cytokine staining assays (ICS) two HIV-1-Nef-derived peptide pools were used to assess HIV-specific T cell responses: one spanning the N-terminal part of HIV-1 Nef (aa 1 to 101) and one spanning the C-terminal part of HIV-1 Nef (aa 96 to 205). These peptide sets were designed as previously described [11] and obtained through the Centre for AIDS Reagents (CFAR) (National Institute for Biological Standards and Control, Hertfordshire, UK). To assess T cell responses we used 15-mer peptides overlapping by 11 amino acids spanning the HBV-core protein (ProImmune Ltd., Oxford, UK), the HCV-NS3 protein, the EBV-BZLF1 protein and the CMV-IE1 protein (JPT Peptide Technologies GmbH, Berlin, Germany).

Three peptides corresponding to optimal epitopes were used to determine epitope-specific T cells in ICS and multimer staining: the HIV-1-Nef-derived HLA-B8 restricted peptide FLKEKGGL (CFAR), the CMV-pp65-derived HLA-A2 restricted peptide NLVPMVATV and the CMV-IE1-derived HLA-A2 restricted peptide VLEETSVML. CMV-peptides as well as MHC-multimers were kindly provided by Prof. D. Busch, Institute for Microbiology, Technische Universität München, Germany.

Stocks of peptides and peptide pools contained between 0.066 and 0.5 mg/ml peptide and were stored at −20°C. Final concentration of DMSO in the assay was lower than 1%.

#### Autologous B-lymphoblastoid cell lines

To generate autologous EBV-transformed B-lymphoblastoid cell lines (B-LCL) PBMC were incubated with supernatants of the EBV secreting cell line B95-8 according to our local SOP. Thereby, B cells are consistently infected with EBV. Cyclosporin A (1 µg/ml) was used to inhibit T cell-mediated killing of infected B cells.

B-LCL present various EBV antigens upon endogenous processing and enable for the assessment of EBV specific T cells. For use in co-culture experiments, autologous B-LCL were mixed with PBMC in a ratio of 1∶2 and subsequently used for ICS.

### Assay and data aquisition

#### In vitro stimulation

For stimulation 10^6^ viable PBMC/well with or without overnight resting were resuspended in 150 µl culture medium containing 2 µg/ml peptide and costimulatory antibodies (1.3 µg/ml anti-CD28 and anti-CD49d, respectively; BD Biosciences, Heidelberg, Germany) in a 96-well polypropylene U-bottom microtiter plate. Whole blood stimulation was carried out respectively in 1 ml of whole blood. Peptide- and mock-stimulated samples were run in parallel to define background. Following 60 min of incubation at 37°C in 5% CO_2_, 10 µg/ml of secretion blocker Brefeldin A (Sigma-Aldrich, Munich, Germany) in a total volume of 50 µl culture medium was added to the cell suspension without mixing and the incubation was carried out for additional 4 hours at 37°C in 5% CO_2_.

#### Intracellular cytokine staining

For whole blood ICS assays red blood cells were lysed after *in vitro* stimulation by adding 9 ml lysing solution (BD Lysing Solution, BD Biosciences) per 1 ml blood and incubating for 10 minutes at room temperature. Remaining lymphocytes were pelleted, resuspended in 100 µl/1 ml blood Perm/Wash buffer (BD Perm/Wash buffer, BD Biosciences) and transferred to a 96-well polypropylene U-bottom microtiter plate. Lymphocytes were permeabilized (BD Permeabilizing solution, BD Biosciences, 500 µl/ml blood), washed twice (BD Perm/Wash buffer, BD Biosciences, 2 ml/1 ml blood) and stained with of the following antibodies (Table 1) in 50 µl Perm/Wash buffer 30 min on ice in the dark.

**Table 1:** Antibodies used for ICS of whole blood.

Antibody	Assay concentration (µg/ml)	Company	Antibody clone	Compensation control
CD154-FITC	1	BD Biosciences	TRAP1	CD8-FITC
MIP1β-PE	0.8	BD Biosciences	D21-1351	CD8-PE
CD4-PerCP	0.3	BD Biosciences	SK3	
CD45RA-PECy7	0.7	BD Biosciences	L48	
CD8-PacB	10	Biozol	DK 25	
CD3-AmCyan	10	BD Biosciences	SK7	
IL2-APC	20	BD Biosciences	5344.111	CD8-APC
IFNγ-Al700	1.6	BD Biosciences	B27	CD3-Al700

For ICS of stimulated PBMC, cells were labelled in 50 µl stain buffer using the LIVE/DEAD® Fixable Near-IR Dead Cell Stain Kit (Invitrogen, Darmstadt, Germany) for 30 minutes (min) on ice in the dark and washed twice with 200 µl FACS buffer (BD Pharmingen Stain Buffer, BD Biosciences). In case of staining of apoptotic cells, PBMC were now surface stained with 2.5 µl Annexin APC (BD Biosciences) in 50 µl FACS buffer for 30 min on ice in the dark and washed twice with 200 µl FACS buffer. PBMC were fixed and permeabilized (BD Cytofix/Cytoperm Kit; BD Biosciences; 100 µl/well) for 20 min on ice in the dark. After washing twice with Perm/Wash solution (BD Cytofix/Cytoperm Kit; BD Biosciences; 200 µl/well) PBMC were stained intracellularly with of the following antibodies (Table 2) in 50 µl Perm/Wash buffer 30 min on ice in the dark. Samples were acquired using a LSRII flow cytometer (BD Biosciences) equipped with a High Throughput Sampler (BD Biosciences) and FACSDiva Software V.5.0 (Becton Dickinson, Heidelberg, Germany). PMT voltages were adjusted on live cells that were apart from live/dead staining unstained for all parameters. Mean autofluorescence values were set to approximately 10^2^ for all used fluorochrome channels. Data analysis was performed using FlowJo version 9.4.10 (Tree Star, Ashland, OR).

**Table 2:** Antibodies used for ICS of PBMC.

Antibody	Assay concentration (µg/ml)	Company	Clone	Compensation control
CD154-FITC	1	BD Biosciences	TRAP1	CD8-FITC
MIP1β-PE	0.8	BD Biosciences	D21-1351	CD8-PE
CD4-PerCP	0.3	BD Biosciences	SK3	
CD45RA-PECy7	0.7	BD Biosciences	L48	
CD8-PacB	10	Biozol	DK 25	
CD3-V500	20	BD Biosciences	SP34-2	
IL2-APC	20	BD Biosciences	5344.111	CD8-APC
TNFα-eFluor450	0.05	eBioscience	MAb11	CD3-eF450
IFNγ-Al700	1.6	BD Biosciences	B27	CD3-Al700

#### MHC-multimer staining

After thawing, PBMC were either rested or used directly for the staining. PBMC were incubated with 2 µg/ml viability dye ethidium monoacide (EMA; Molecular Probes, Leiden, The Netherlands) in 50 µl FACS buffer for 20 min on ice in the dark. After washing twice with 200 µl FACS buffer, cells were stained with the respective PE-conjugated multimer at a concentration of 1.85 µg/ml for 30 minutes on ice in the dark, washed again twice and fixed for 20 min (BD Cytofix solution; BD Biosciences; 200 µl/well) with subsequent washing twice. PBMC were than stained with CD3-HorizonV500 (BD Biosciences) and CD8-PacB (Biozol) in 50 µl FACS buffer. Antibodies have been pre-titered and optimal concentration for extracellular staining has been used.

#### Cytometric bead array

BD Cytometric Bead Array Flex Sets (BD Biosciences) were used according to the manufacturer's instructions. IFNγ, TNFα, IL1β, IL6, IL2, IL10, MIP1α, MIP1β and Rantes were quantified in the culture supernatant at different time points during the resting period. Standard curves for each cytokine were generated using the provided premixed lyophilized standards. Data was analysed with FCAP Array (version 3.0.1; Softflow, Burnsville, MN).

#### T cell isolation

T cells were isolated from PBMC by magnetic activated cell sorting (MACS technique) using Pan T Cell Isolation Kit (Miltenyi Biotec, Bergisch-Gladbach, Germany) according to the manufacturer's instructions. Non-T cells are indirectly magnetically labelled by using a cocktail of biotin-conjugated antibodies against CD11b, CD16, CD20, CD56, and CD66abce, and anti-Biotin MicroBeads. Isolation of untouched T cells revealed a purity of more than 95%.

### Data analysis and interpretation

#### Gating Strategy

Gating strategy is shown in the supplementary information (figure S1). Each gate was designed in the negative control sample with consideration of marker downregulation from stimulated samples. Gating has been controlled by at least two independent audits. According to the differential expression of CD45RA, CD154, IFNγ, TNFα, IL2 and MIP1β 62 responding CD4 and 30 responding CD8 T-cell subpopulations were identified (CD154 expression as not considered for CD8 T-cell responses). An individual threshold level for each subpopulation was calculated following background subtraction using SPICE software version 4 [12]. This is important for correct data evaluation as the nonspecific background becomes very low or even zero when examining combinations of 3 or more positive functions. The threshold level was defined for each functional combination as the 90^th^ percentile of the distribution of negative values from 161 stimulated samples derived from 32 individuals, including the study participants. Values lower than the respective individual threshold level were set to 0. Furthermore, a general threshold of 0.005% was applied for all CD8 and CD4 T-cell subsets to exclude minor responses. As a result threshold was ranging from 0.005 to 0.183% for CD8 T cell analysis and 0.005 to 0.167% for CD4 T cell analysis, according to the different subpopulations of responding T cells. Functional profiles of antigen specific T cells are reported as pie charts, depicting the contribution of responding T-cell subpopulations to the total antigen specific response according to their functionality (mono- to poly-functional) using SPICE software version 4 [12]. Subpopulations with four functions are shown in red, with three functions in green, bifunctional cells are shown in blue and monofunctional cells in grey. Color coding used to generate pie charts is matched within all figures to allow direct comparison of the relative size of each subpopulation of antigen specific T cells between different experiments.

#### Statistical analysis

Nonparametric statistical tests were applied in all cases. Paired Wilcoxon signed rank tests were used to assess significance of change in values between experiment conditions. A confidence interval of 95% was used for all statistical considerations (GraphPadPrism version 5.01, GraphPad Software Inc., San Diego, CA).

Generated raw data can be provided upon request.

### Laboratory environment

This study was performed by trained stuff (work experience: 2–6 years) in our laboratory which does not operate under GLP conditions. Established SOP were followed covering the processing, freezing, storage, and thawing of PBMC as well as the stimulation and staining procedure, data acquisition, gating strategy and data processing. The assays were performed by the same individuals throughout the course of the study.

## Results

### Overnight resting decreases post-thaw cell viability of PBMC

**Figure 1:**
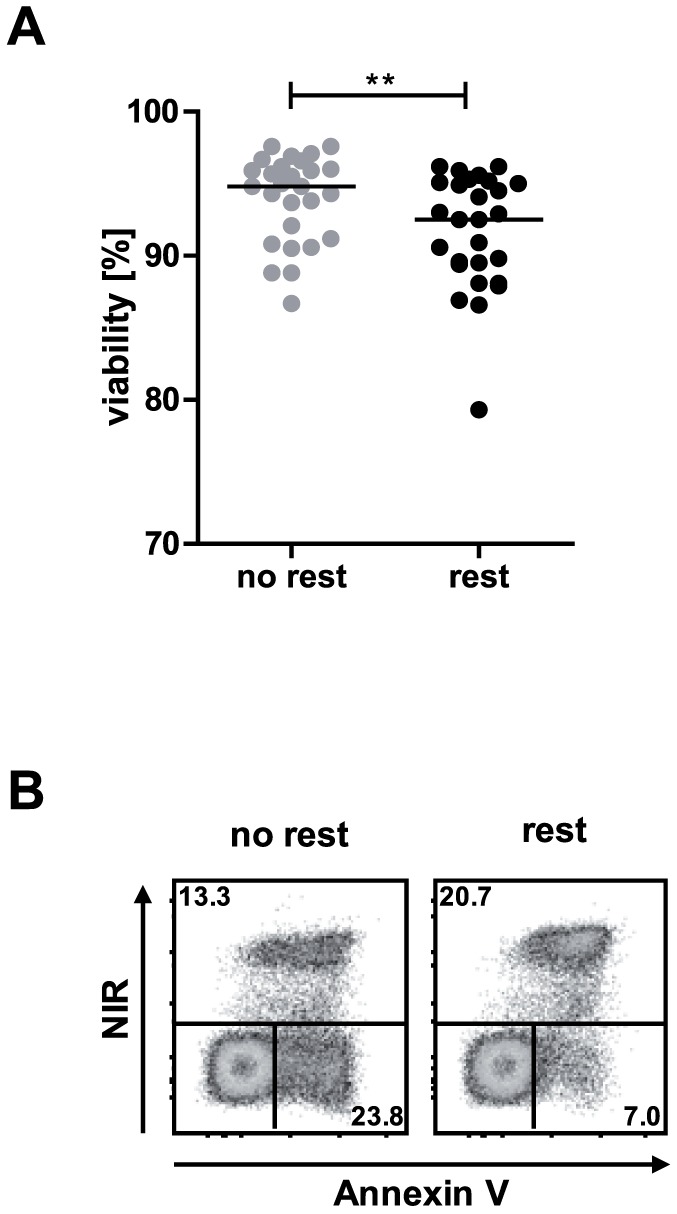
Overnight resting reduces post-thaw viability of PBMC. (A) Flow cytometric analysis of lymphocyte viability compared between not rested and rested cells in a cohort of 21 individuals (** p<0.005, Wilcoxon matched pairs test; median indicated by black line). (B) Flow cytometric live/dead (NIR-/NIR+) discrimination and costaining with Annexin V for resolution between dead (NIR+) and apoptotic (NIR-/Annexin V+) cells in not rested and rested PBMC. NIR: near-infrared.

To determine effects of overnight resting we used PBMC of a cohort consisting of 21 individuals with different chronic (HIV, HBV, HCV) or persistent (EBV, CMV) infection. First we determined the influence of overnight resting on cell viability. Cell viability significantly decreased (p = 0.0013) after overnight resting of PBMC with a median of 94.8% (range: 86.7–97.6%) and 92.5.% (range: 79.3–96.2%) viable lymphocytes for not rested and rested PBMC, respectively (figure 1A). Staining for the apoptosis marker Annexin V revealed that PBMC without rest contained a higher percentage of apoptotic cells (NIR-/Annexin V+), whereas rested PBMC contained a higher percentage of dead cells (NIR+) (figure 1B). This indicated that overnight resting enables apoptosis-prone cells to die resulting in a PBMC population with a higher numbers of truly viable cells.

### Overnight resting significantly increases the quantity and changes the functional profile of antiviral CD8 and CD4 T-cell responses

To test whether overnight resting affects quantity and quality of antiviral T-cell responses we determined CD8 and CD4 T cells specific for HIV Nef, HCV NS3, HBV core, EBV BZLF-1 and CMV IE1 antigens in our cohort *ex vivo*.

**Figure 2:**
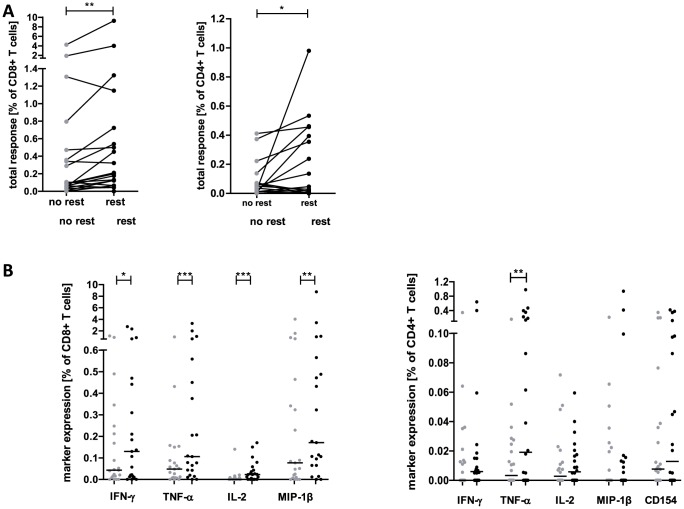
Overnight resting increases the magnitude of antiviral T-cell responses detectable by ICS. (A) Comparison of total numbers of functional CD8 (left panel) and CD4 (right panel) T cells specific for HIV, HBV, HCV and EBV in a cohort of 21 individuals. (B) Single marker expression of antiviral CD8 (left panel) and CD4 (right panel) T cells. All analyses were performed on not rested and rested PBMC as indicated. Background values as determined in unstimulated controls are subtracted and a predefined threshold on subpopulation level is applied before calculating the total response and amount of single cytokines (see materials and methods). (*** p≤0.001; ** p≤0.05; * p≤0.05, Wilcoxon matched pairs test; median indicated by black line).

For all antigen specificities, overnight resting increased the overall frequency of responding antigen specific CD8 T cells from 0.09% (range: 0–4.24%) to 0.20% (range: 0–9.27%; [p = 0.005]) (figure 2A, left). Overall antiviral CD4 T-cell responses increased only slightly (p = 0.045) when comparing not rested versus rested PBMC (0.02%; range: 0–0.41% and 0.02%; range: 0–0.98%, respectively) (figure 2A, right). In antigen specific CD8 T cells expression of all four functional markers increased significantly (IFNγ (p = 0.0186), IL2 (p = 0.0001), TNFα (p = 0.0006) and MIP1β (p = 0.0014); figure 2B, left), whereas only TNFα expression (p = 0.0066) increased in CD4 T cells after overnight resting (figure 2B, right).

**Figure 3:**
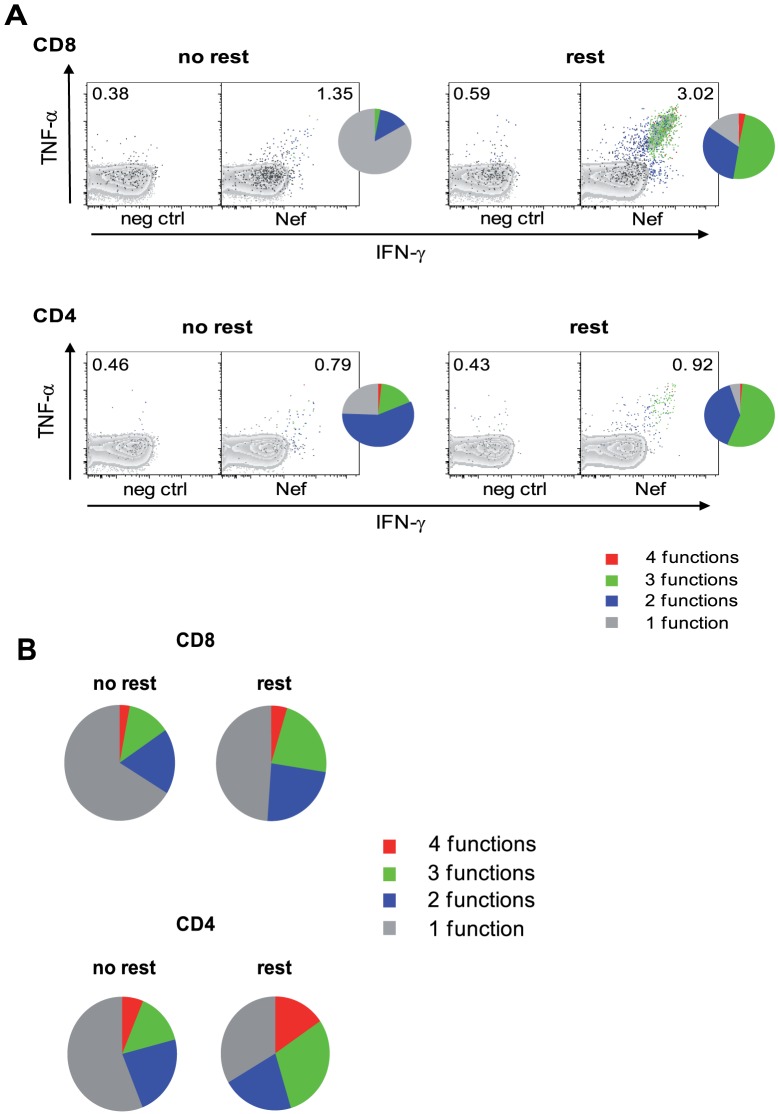
Overnight resting increases functionality of antiviral T cells. (A) HIV Nef specific CD8 (upper panel) and CD4 (lower panel) T cells were determined by ICS. Unstimulated PBMC served as negative controls (neg ctrl). Dot plots show functional T cells with numbers as% of total CD8 and CD4 T cells, respectively. Pie charts show the relative contribution of each T-cell subpopulation within the total HIV Nef specific T-cell response according to their functionality (mono- to poly-functional). One representative experiment is shown. (B) Pie charts represent the relative functional composition of total HIV, HBV, HCV and EBV specific CD8 (upper panel) and CD4 (lower panel) T-cell responses in a cohort of 21 individuals. All analyses were performed on not rested and rested PBMC as indicated.

Next we analyzed whether overnight resting also affects the quality (i.e. functional profile) of antigen specific CD8 and CD4 T-cell responses detectable *ex vivo*. As exemplarily shown for an HIV Nef specific T-cell response overnight resting also impacts the functional profile of antigen specific CD8 and CD4 T cells (figure 3A). Monofunctional CD8 and CD4 T cells, producing only one cytokine or chemokine decreased and a more polyfunctional profile was observed after overnight resting for all tested antigen-specificities (figure 3B). The fraction of monofunctional cells clearly decreased upon resting (CD8 T cells: 66.2% to 48.8%, p = 0.0011; CD4 T cells: 56.4% to 33.5%, not significant) with simultaneous significant increase of polyfunctional T cells (CD8 T cells: increase of T cells with 3 (p = 0.0112) and 4 (p>0.0001) functions, respectively; trifunctional CD4 T cells, p = 0.034). To determine the time dependency of the resting effect we directly compared HIV specific CD8 T-cell responses of PBMC rested between 1 and 24 hours. Dependant on the resting period CD8 T cells showed a gradual increase in quantity and functionality maxing out already at 18 hours (figure S2). A resting period of twenty-four hour did not further increase the resting effect.

These results showed that overnight resting of PBMC increased numbers and functionality of antigen specific CD8 and CD4 T cells *ex vivo* detectable by ICS.

### Overnight resting increases antigen sensitivity of CD8 T cells

**Figure 4:**
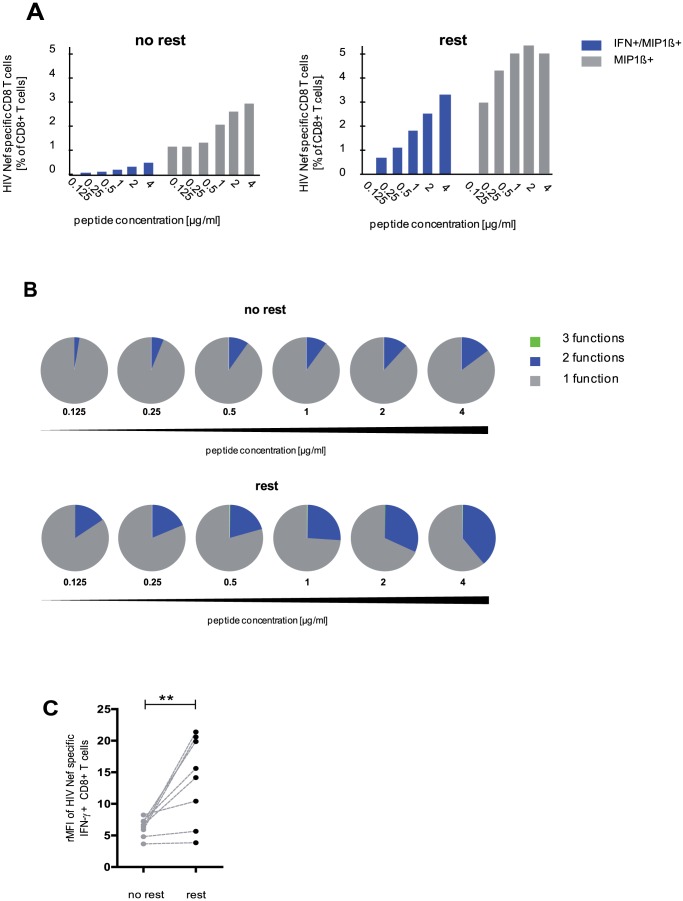
Overnight resting heightens sensitivity to antigens. (A) Bar charts indicate frequencies of bi- and mono-functional (blue: IFN-γ+/MIP-1β+; grey: MIP-1β+) HIV Nef specific CD8 T cells for a given peptide concentration (0.125–4 µg/ml) as percentage of total CD8 T cells. Cells were tested without (upper panel) or with (lower panel) overnight resting. (B) Pie charts show the functional profiles of HIV Nef specific CD8 T cells for a given peptide concentration. Cells were tested without (upper panel) or with (lower panel) overnight resting. (A) and (B) one representative experiment is shown. (C) Relative mean fluorescent intensity (rMFI) of IFN-γ staining of HIV Nef specific CD8 T cells of eight individuals normalized to rMFI of total CD3 T cells. All analyses were performed on not rested and rested PBMC as indicated. (** p<0.005, Wilcoxon matched pairs test; median indicated by black line).

To identify possible effects of overnight resting on antigen sensitivity we tested rested versus not rested CD8 T cells in peptide titration assays. As shown for HIV Nef specific CD8 T cells, progressive increases in peptide concentrations stimulated higher numbers of antigen specific T cells (figure 4A). At any given peptide concentration, a greater proportion of the rested CD8 T-cells was bifunctional compared to their not rested counterparts (figure 4B). Non-rested PBMC were predominantly monofunctional (97.4% MIP1β+) with only a minor bifunctional fraction (2.6% IFNγ+/MIP1β+) using the lowest antigen concentration (figure 4B, upper panel). Stimulation at higher peptide concentrations resulted in an increasing fraction of bifunctional T cells (IFNγ+/MIP1β+) up to 11.7% of all HIV Nef specific CD8 T cells. Rested PBMC were IFNγ+/MIP1β+, already at the lowest peptide concentration to 15.3% of all HIV Nef specific CD8 T cells expanding to 31.7% at the highest peptide concentration (figure 4B, lower panel). In addition to their higher functional avidity rested PBMC produce more cytokines on a per cell basis compared with not rested cells as indicated by the relative mean fluorescence intensity (rMFI) of a single cell. The rMFI of IFN-γ expressing HIV Nef specific CD8 T cells was clearly enhanced in rested compared to not rested cells (p = 0.008; median 14.9 and 6.5, respectively) (figure 4C). The same applied to rMFI of MIP-1β (data not shown). These data indicate that overnight resting can increase functional avidity of antigen specific CD8 T cells enabling a more sensitive detection of antigen specific CD8 T cells *ex vivo*.

### Overnight resting does not influence the total number of epitope specific CD8 T cells

**Figure 5:**
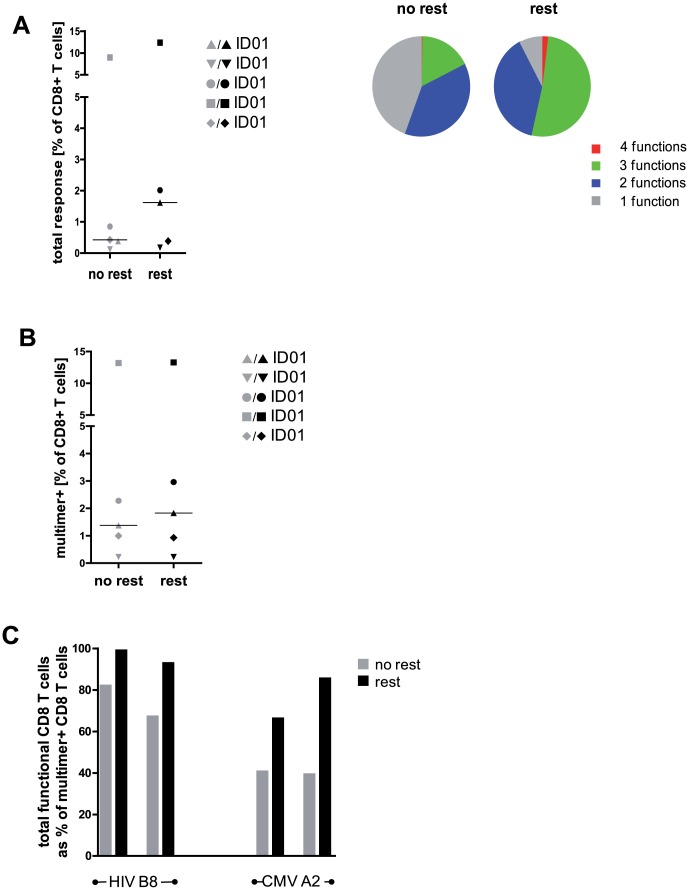
Overnight resting does not alter the total number of epitope specific T cells. (A) Frequencies (left panel) and functionality (right panel) of HIV and CMV specific CD8 T cells were determined by ICS. Data indicate numbers of functional T cells as% of total CD8 T cells. Pie charts show the relative contribution of each functional subpopulation within the total CD8 T-cell response according to their functionality. (B) Frequencies of HIV and CMV specific CD8 T cells determined by multimer staining. Data indicate numbers of multimer-positive T cells as% of total CD8 T cells. (A) and (B): data from five HIV (n = 3) or CMV (n = 2) seropositive individuals are shown; median indicated by black line. (C) Comparison of T cell ICS and multimer staining. Bar charts show frequencies of functional epitope specific CD8 T cells determined by ICS calculated as percentage of corresponding multimer-positive T cells. Representative results of two individuals with known epitope specificities (HLA-B8 restricted HIV Nef and HLA-A2 restricted CMV IE1, respectively) are shown. All analyses were performed on not rested and rested PBMC as indicated.

To test whether overnight resting increases the total detectable numbers or the functionality of antigen specific CD8 T cells we performed a multimer staining in parallel to ICS using optimal CMV and HIV derived epitopes with known HLA restriction. MHC multimer staining allowed determining the number of T cells specific for a given epitope irrespective of their functionality. Overnight resting increased the percentage of functional, epitope specific CD8 T cells detectable by ICS (figure 5A, left panel) and T cells showed a more polyfunctional profile (figure 5A, right panel). On the contrary, the total number of detectable antigen specific CD8 T cells determined by multimer staining remained unchanged independent of assay conditions (figure 5B). We compared numbers of functional CD8 T cells of two individuals determined by ICS with the corresponding results of multimer staining (figure 5C). For both epitope specificities (HLA-B8 restricted HIV Nef and HLA-A2 restricted CMV IE1, respectively) rested PBMC closer reflected the results obtained by multimer staining.

These results demonstrate that overnight resting of PBMC increased numbers of functional CD8 T cells *ex vivo* detectable by ICS but did not affect total numbers of antigen specific CD8 T cells detectable by multimer staining.

### “Resting effect” is not mediated by antigen presenting cells

**Figure 6:**
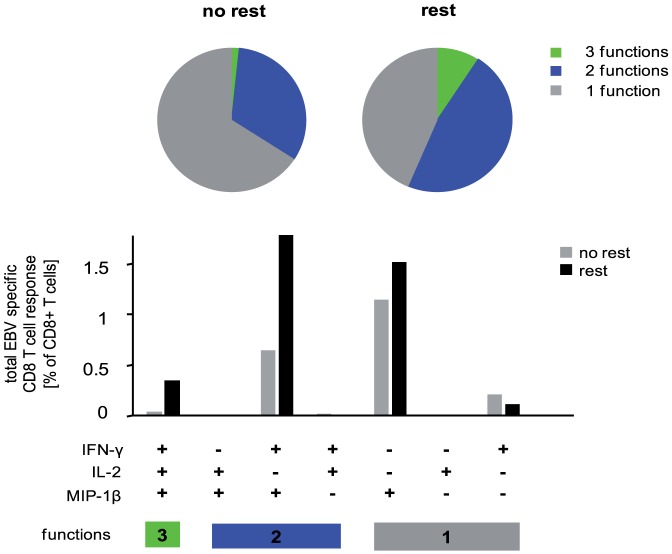
Resting effect is not mediated by antigen presenting cells. (A) Autologous EBV-transformed B-lymphoblastoid cell lines were used as antigen presenting cells to stimulate antigen specific T cells of an EBV seropositive subject. PBMC without the addition of EBV-transformed B-lymphoblastoid cell lines provided the negative controls used for background subtraction. (B) CD3 T cells of a CMV seropositive subject were isolated by magnetic cell sorting. After cryopreservation, CD3 T cells were stimulated directly or after overnight rest with a pool of overlapping peptides corresponding to the CMV-IE-1 protein. (A) and (B) IFNγ, IL2 and MIP1β production was determined by ICS directly or after an overnight resting. Frequencies (bar charts) and functional composition (pie charts) of EBV (A) and CMV (B) specific CD8 T cells are shown. CD8 T-cell subpopulations are depicted according to their functionality (three functions: green; two functions: blue; monofunctional cells: grey). One representative experiment is shown.

The effect of overnight resting, namely increased frequency and functional avidity and a more polyfunctional profile of antigen specific T cells is subsequently termed “resting effect”. To investigate if the “resting effect” is mediated by antigen presenting cells, we used autologous EBV-transformed B-lymphoblastoid cell lines (B-LCL) as perpetual EBV-antigen presenters. B-LCL were used in combination with rested or non-rested PBMC of an EBV seropositive individual. Despite the use of identical antigen presentation we still determined higher frequencies and increased functionality of EBV specific CD8 T cells upon resting (figure 6).

In order to determine whether overnight resting influences T cells rather than non T cells, isolated CD3+ T cells were rested or not rested prior to loading with a CMV IE-1 derived peptide pool. The resting effect still could be observed (figure 6B), indicating that the resting procedure affects T cells rather than other cell types. These findings indicated that the “resting effect” is not mediated by an altered function of antigen presenting cells, but suggested that overnight resting rather directly affects T cells.

### Overnight resting induces release of proinflammatory cytokines and chemokines

**Figure 7:**
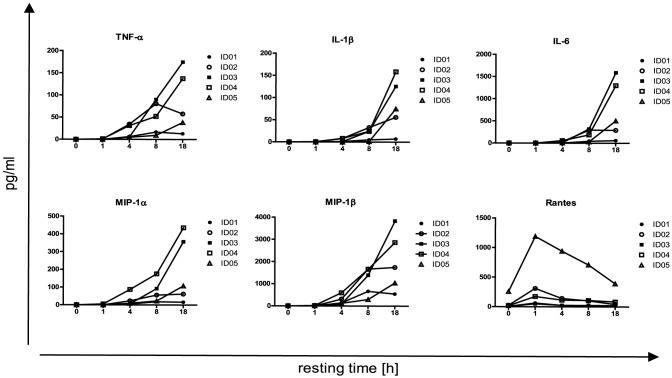
Overnight resting triggers production of proinflammatory cytokines and chemokines. (A) Concentration of proinflammatory cytokines (TNFα, IL1β, IL6,) and chemokines (MIP1α, MIP1β, Rantes) in supernatants of unstimulated PBMC from five subjects were determined by cytometric bead array at indicated time points during the resting period of 18 hours. One representative experiment is shown.

Next, we performed a cytometric bead array to quantify overall cytokine and chemokine production at several time points during the resting period. PBMC of three healthy and two HIV infected individuals were used to simultaneously assess concentrations of secreted cytokines TNFα, IL1β, IL6, IFNγ, IL2 and IL10 as well as the chemokines MIP1α, MIP1β and Rantes. IFNγ, IL2 and IL10 were not detectable at any time point. Rantes was produced already after 1 hour of resting. After 4 hours of resting, a release of TNFα, IL1β, IL6 as well as MIP1α and MIP1β became detectable and further increased over time (figure 7). Because these data suggested that cytokine and chemokine production during the resting period might contribute to the observed “resting effect” we cultured freshly thawed PBMC with preconditioned medium of rested cells for 1–24 h prior to stimulation (S2C). Functionality did not recover faster compared to standard resting conditions (figure S2B).

### Overnight resting “rescues” T-cell responses of cryopreserved PBMC

Finally the findings of our study raised the question which experimental procedure corresponds best to the *in vivo* situation of a T cell encountering its antigen. ICS of whole blood samples after *ex vivo* restimulation can be considered the least artificial assay. We compared this procedure with ICS assays using rested or not rested freshly isolated or cryopreserved PBMC.

**Figure 8:**
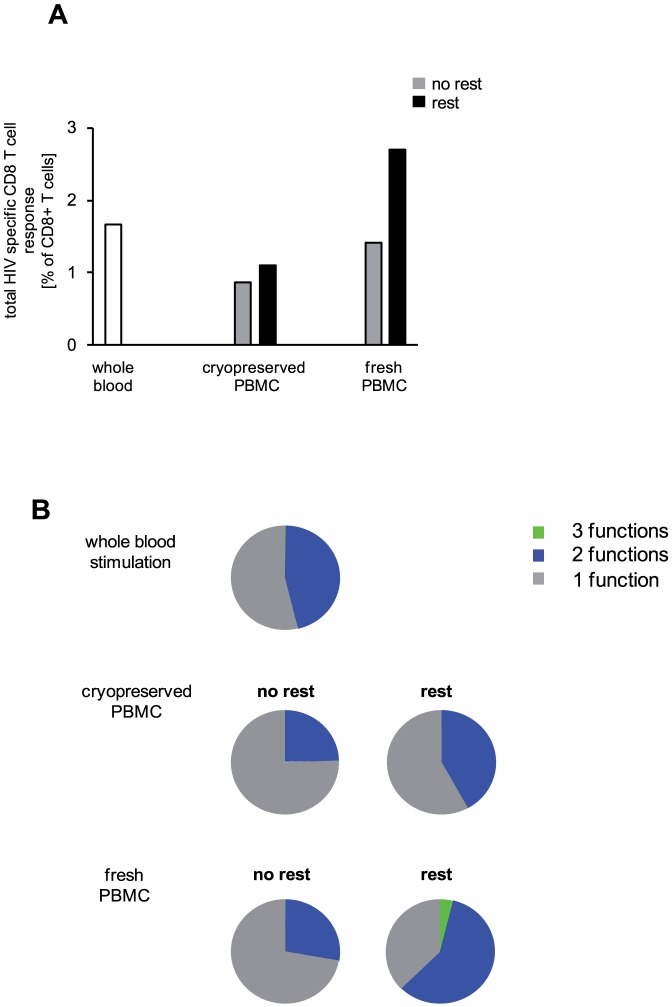
Overnight resting rescues CD8 T-cell responses of cryopreserved PBMC. T-cell responses of an HIV infected patient were determined after whole blood stimulation and after stimulation of freshly isolated PBMC or cryopreserved PBMC of an HIV infected subject. IFNγ, IL2 and MIP1β production was determined by ICS. (A) Frequencies and (B) functional composition of HIV Nef specific CD8 T cells are shown. T-cell subpopulations are depicted according to their functionality (three functions: green; two functions: blue; monofunctional cells: grey). Analysis for (A) and (B) were performed on non-rested and rested PBMC as indicated. One representative experiment is shown.

Using whole blood *ex vivo* stimulation we detected a total number of 1.67% HIV specific CD8 T cells (figure 8A). Trifunctional cells were almost undetectable, whereas bifunctional cells had a relative contribution of 45.6% to the total response (figure 8B, upper panel). T-cell responses measured using cryopreserved PBMC without rest were clearly lower with a less functional profile (total CD8 T-cell response: 0.87% with 24.7% bifunctional cells) (figure 8A and 8B, middle panel). Upon resting frequency and functionality increased as expected (total CD8 T-cell response: 1.06% with 42.1% bifunctional cells) reaching values detected in whole blood ICS.

Frequencies of HIV specific CD8 T cells detectable using freshly isolated PBMC were similar to those obtained by whole blood ICS whereas their grade of functionality was slightly lower (total CD8 T-cell response: 1.41% with 27.4% of bifunctional cells) (figure 8A and 8B, lower panel). Overnight resting of freshly isolated PBMC resulted in T-cell responses above those determined in whole blood (total CD8 T-cell response: 2.70% with 58.9% bifunctional and 4.0% trifunctional cells). Whether these additionally detectable cells are active *in vivo* or represent a population with an artificially increased functional phenotype remains to be addressed. Taken together, overnight resting of cryopreserved PBMC increased T-cell responses to levels detected in whole blood directly ex vivo.

## Discussion

Currently numerous antiviral vaccine candidates and cancer immunotherapies designed to elicit specific T-cell responses are evaluated in clinical trials. Reliable and sensitive assays are needed to assure correct measurement and to validate T-cell immunogenicity as correlate of vaccine mediated protection. Clear definitions of immune signatures of effective T cells are therefore required and assays measuring immunogenicity need to be standardized and validated to guarantee verity of results as well as comparability between laboratories. Simultaneous production of multiple cytokines and expression of certain phenotypic markers by either CD4 or CD8 T cells is proposed to represent a correlate of vaccine mediated protection against various infectious diseases [5], [13], [14]. Measuring such functional signatures of T-cell responses represent a useful tool to monitor virus-associated disease activity and there is a clear need for substantial development to validate immune biomarker assays for the application in clinical diagnostic laboratories [15].

Several parameters of an experimental procedure can influence the outcome of an immune biomarker assay and the present study clearly showed that overnight resting of PBMC at 37°C prior to *ex vivo* restimulation is a procedure with a very high impact on the results of T-cell assays in terms of detectable numbers as well as functional profile of T cells.

Previous studies including investigations of overnight resting and its effect on T-cell immune responses were limited to HIV and CMV specificities [4], [6], [16]. We broadened the analysis considering EBV as well as HBV and HCV specific T-cell responses. Our investigation included assessment of multiple T-cell markers (i.e. cytokine and chemokine production, expression of phenotypic markers and activation marker) and their simultaneous expression.

Higher numbers of responding antigen specific T cells with a higher grade of functionality were detectable in *ex vivo* ICS assays when PBMC were rested prior to peptide restimulation. Increased functionality of T cells after resting matched the observation of a significantly higher relative mean fluorescence intensity (rMFI) for IFN-γ, corresponding to more production of this cytokine on a per cell level [2], [13]. High rMFI values of cytokine stainings which correspond to a bright staining allow for clear distinction between positive and negative populations. Thus overnight resting can also simplify the analysis of complex flow cytometric data.

In parallel with increased functionality, functional avidity of T cells was clearly augmented by overnight resting in a time dependent process. This finding confirms other studies demonstrating in detail that polyfunctionality is a correlate of antigen sensitivity [17]. Functional avidity of T cells is impacted by several factors (e.g T cell receptor (TCR) affinity, expression levels of TCR and co-receptors and availability of different signaling molecules) [18]. We did not investigate the effect of overnight rest on these factors but we assume that the gain of functionality is related to the observed lower activation threshold of the corresponding T cells. Importantly the total T-cell frequencies for all tested antigen-specificities were not influenced by the resting procedure as shown by multimer stainings.

To our knowledge possible mechanisms how overnight resting could affect T-cell immune responses have not yet been investigated in detail. One current opinion is that the resting period enables removal of apoptotic cells and allows cells to recover fully functional ability [5]. Our results confirm this hypothesis as we observed a reduced viability after overnight resting accompanied with lower numbers of apoptotic cells. Our results also raised the question whether the resting effect is subject to a certain lymphocyte population. Using a perpetual source of APC or isolated T cells the resting effect was still detectable suggesting that it is largely mediated by T cells rather than by APC.

An interesting finding of the present study lies in the detected release of proinflammatory cytokines and chemokines during overnight resting. Potentially, proinflammatory cytokines could function as a third signal provided to T cells besides TCR stimulation and costimulatory molecules on antigen presenting cells (APC) [19]. When binding to their receptors, proinflammatory cytokines as well as chemokines could lead to a pre-activation of T cells ahead of peptide restimulation. As preconditioned medium added prior to stimulation did not accelerate the gain of T cell functionality we assume that the resting effect is mediated not only by the cytokine milieu but also via a direct cell-cell contact.

An additional hypothetical mechanism which could serve as an explanation for the “resting effect” could be a pre-activation of T cells via non-cognate CD8-peptide/MHC interaction during resting. Even during an immune response only a very small proportion of the peptide–MHC complexes that are expressed on the surface of an APC is likely antigenic for a given T-cell receptor and most of the peptides that are presented by MHC molecules are non-stimulatory peptides [20]. It has been shown that such non-stimulatory peptides contribute to T-cell activation and immunological synapse formation [21], [22].

Our data so far suggest, that several factors contribute to a pre-activation of T cells during the resting period and a causal relation remains speculative and needs further investigation and verification. Nevertheless the pre-activation hypothesis would match the finding of increased antigen sensitivity and polyfunctionality upon overnight resting. A pre-activation could also explain the higher functionality of T cells in freshly isolated, rested PBMC when compared to a whole blood stimulation assay. When considering all different aspects of our study the question has to be asked, if resting of PBMC should be introduced in an experimental procedure for functional T-cell assays. Since 2005, two consortia have performed proficiency panel experiments to address assay harmonization and as a consequence the MIATA (“*Minimum Information About T-cell Assays*”) initiative was launched [9], [10], [23]. Investigating antigen specific T cells as a primary endpoint in clinical trials requires highly sensitive and reproducible assays to determine T cell reactivity *ex vivo* correlating with clinical efficacy.

The proinflammatory milieu developing during resting of PBMC as well as the dense contact and a possible stimulation via non-cognate peptide-MHC complexes might represent an artificial compound in the experiment not mimicking the *in vivo* situation of antigen encounter in the periphery. However the majority of specific T cells carry out their function primarily in infected tissues, where one could expect a pro-inflammatory milieu and high density of cells.

However, when using cryopreserved PBMC for functional T-cell assays, responses can be lost as shown by our data and others [16]. When using cryopreserved PBMC overnight resting can provide an opportunity to rescue T-cell responses otherwise undetectable. Hence, resting can be of use for conduction of clinical studies, i.e. multicenter trials where the use of frozen PBMC is unavoidable. Our direct comparison of specific T cells measured by ICS versus multimer staining demonstrated clearly that by overnight resting, frequencies of functional T cells as measured by ICS can be increased corresponding to values of multimer-positive antigen specific T cells. Because the number of functions that can be measured simultaneously by multicolor flow cytometry is limited, the risk remains that antigen specific T cells are missed because functional markers of interest are not included in the staining. By introducing a resting period into the experimental procedure of an ICS assay, traceability of low grade functional T cells which are common in chronic viral infection can be enhanced. Overnight resting can hence provide an improved experimental setup increasing sensitivity of functional T-cell assays.

Taken together, investigators have to decide for each experimental setting and clinical trial if overnight resting should be used for any immune biomarker assay. Our data suggest that it can be of use to increase sensitivity of functional T-cell assays. Care has to be taken when interpreting results of functional T-cell assays and when comparing data from different laboratories using different experimental protocols. We clearly showed that overnight resting has a prominent impact on perceived frequency and functionality of T cells which precludes direct comparison between studies differing in this experiment modification. It is therefore of utmost importance to maintain assay conditions throughout a study and to report the experimental setup which is used. The knowledge gained in the present study is crucial for optimization and standardization of immune monitoring of clinical trials and to guarantee comparability of results generated in different laboratories.

## Supporting Information

Figure S1 **Gating strategy.** Lymphocytes were gated based on FSC versus SSC plot (A), followed by exclusion of dead cells by NIR staining (B). As representatively shown, we gated for CD3 cells on all functional markers to account for CD3 downregulation on antigen specific responding T cells and combined these gates with the Boolean operator “OR” to obtain the CD3 cell population (C). We gated for CD4 and CD8 cells on all functional markers to account for downregulation on antigen specific responding T cells and combined these gates with the Boolean operator “OR” to obtain the CD4 and CD8 cell population (D and E, respectively). CD4 T cells were excluded from the CD8 T-cell population and vice versa (F). Once CD4 and CD8 T cell population was defined, CD4 T cells (G-L) positive for IFNγ, TNFα, IL2, MIP1β, CD154 and CD45RA and CD8 T cells (M-Q) positive for IFNγ, TNFα, IL2, MIP1β and CD45RA were separately identified by using different plots in which the axis were chosen to provide the best discrimination between positive and negative events Selection of positive cells for the functional markers was done by comparison with a mock-stimulated sample.
(EPS)

Figure S2 **Effect of overnight resting on T cells is time dependent.** PBMC of an HIV infected subject were stimulated without rest (A) or rested before stimulation for 1–24 hours (B and C) in culture medium (B) or in supernatant of 18 hours rested PBMC (C). Stimulation has been performed using an HLA-B8 restricted HIV Nef-derived epitope. IFNγ, IL2, TNFα and MIP1β production was determined by ICS. Pie charts represent the functional composition HIV-nef specific CD8 T cells. CD8 T-cell subpopulations are depicted according to their functionality (four functions: red; three functions: green; two functions: blue; monofunctional cells: grey).
(EPS)
